# Transient elastography in patients at risk of liver fibrosis in primary care: a follow-up study over 54 months

**DOI:** 10.3399/BJGPO.2021.0145

**Published:** 2021-11-24

**Authors:** Tina Reinson, Christopher D Byrne, Janisha Patel, Magdy El-Gohary, Michael Moore

**Affiliations:** 1 Researcher, Primary Care, Population Sciences and Medical Education, Faculty of Medicine, University of Southampton, Southampton, UK; 2 Professor of Endocrinology and Metabolism, Human Development and Health, Faculty of Medicine, University of Southampton, Southampton, UK; 3 Consultant Hepatologist, Department of Gastroenterology and Hepatology, University Hospital Southampton, Southampton, UK; 4 GP, Shirley Health Partnership, Southampton, UK; 5 Professor of Primary Health Care Research, Primary Care, Population Sciences and Medical Education, Faculty of Medicine, University of Southampton, Southampton, UK

**Keywords:** primary healthcare, mass screening, early diagnosis, risk reduction behaviour, liver diseases, general practice

## Abstract

**Background:**

Liver fibrosis assessment services using transient elastography are growing in primary care. These services identify patients requiring specialist referral for liver fibrosis, and provide an opportunity for recommending lifestyle change. However, there are uncertainties regarding service design, effectiveness of advice given, and frequency of follow-up.

**Aim:**

To assess the following: (a) effectiveness of standard care lifestyle advice for weight management and alcohol consumption; (b) uptake for liver rescan; and (c) usefulness of a 4.5-year time interval of rescanning in monitoring progression of liver fibrosis.

**Design & setting:**

Analysis of patient outcomes 4.5 years after the first ‘liver service’ attendance that included transient elastography in five GP practices in Southampton, UK.

**Method:**

Outcomes included weight, alcohol consumption, rescan uptake, time interval between scans, and change in liver fibrosis stage.

**Results:**

A total of 401 participants were recontacted. Mean standard deviation (± SD) weight loss was 1.2 kg±8.4 kg (*P* = 0.005); Alcohol Use Disorders Identification Test (AUDIT) grade increased by 7.8% (*P* ≤0.001). A total of *n* = 116/401 participants were eligible for liver rescanning and *n* = 59/116 (50.9%) agreed to undergo rescanning. Mean ± SD time interval between scans was 53.6±3.4 months. Liver fibrosis progressed from mild (≥6.0 kPa–8.1 kPa) to significant fibrosis (8.2 kPa–9.6 kPa) in 3.4% of patients; from mild to advanced fibrosis (9.7 kPa–13.5 kPa) and cirrhosis (≥13.6 kPa) in 15.3% of patients, and did not progress in 81.3%. No baseline factors were independently associated with liver fibrosis progression at follow-up.

**Conclusion:**

Rescan recall attendance and adherence to lifestyle changes needs improving. Optimum time interval between scans remains uncertain. After a mean interval of 53.6 months between scans, and with no specific predictors indicated, a substantial minority (18.7%) experienced a deterioration in fibrosis grade.

## How this fits in

Being overweight or obese and drinking above the recommended weekly units of alcohol are two of the main risk factors for the development and progression of liver disease. Losing weight and/or drinking less alcohol will improve liver health as well as overall health. Transient elastography is being used in primary care to scan patients and identify liver fibrosis and cirrhosis. However, there are uncertainties regarding patient uptake for liver rescanning, the ideal time interval for a follow-up liver rescan to enable identification of progressive liver fibrosis, and the effectiveness of standard care advice for weight management and alcohol consumption. The aims were to assess whether standard care advice to lose weight and reduce alcohol consumption was effective in: (a) restoring and maintaining ideal body weight; and (b) moderating alcohol consumption. Additionally, the study aimed to determine what proportion of participants would experience a progression in liver fibrosis stage at follow-up liver scan.

## Introduction

The estimated annual cost of liver disease is £5.24 billion,^1^ and it is the third biggest cause of premature mortality.^2^ The principal cause of liver disease is excess alcohol consumption;^3^ however, 30% of the UK population have non-alcoholic fatty liver disease (NAFLD),^4^ which is often undiagnosed,^2^ and can progress to cirrhosis, liver failure, or liver cancer; poor quality of life; and death.^1^ Liver disease places a huge burden on the NHS in terms of costs and resource utilisation, both of which are predicted to increase.^1,5^ Besides increasing risk of liver morbidity and mortality, NAFLD is a multisystem disease^6^ that also increases risk of extra-hepatic diseases such as cancer, type two diabetes (T2DM), cardiovascular disease, and chronic kidney disease.^7,8^

Around three-quarters of patients with cirrhosis remain undetected until they present as an emergency with the complication of advanced liver disease, and only one-third survive in the long term.^2,3,9^ Detection of liver disease is difficult because it progresses silently with no signs or symptoms until liver failure develops^10^ and the opportunity for intervention is missed. In 2020, the National Institute for Health and Care Excellence (NICE) recommended the use of vibration-controlled transient elastography^11^ (VCTE) for assessing liver fibrosis and cirrhosis in primary care.^12^

Local care and treatment of liver disease (LOCATE) was a large feasibility trial that embedded specialist liver nurses into GP surgeries.^13^ The primary objective of LOCATE was to evaluate whether using the combined results of VCTE and liver fibrosis markers^14^ would, when compared with usual care, improve the identification of liver fibrosis.

Secondary to identifying liver disease within the community, the LOCATE intervention also provided patients with a brief behavioural intervention (BI) at the time of their liver health assessment. The BI was delivered by specialist liver nurses who would inform patients of their VCTE reading (Supplementary Box 1), and offer appropriate lifestyle changes regarding weight management (Supplementary Box 2) and alcohol consumption (Supplementary Box 3).

### Aims

To report on an ‘at-risk’ group of patients managed solely in primary care:

1. Whether the standard care advice to lose weight and drink within UK alcohol unit guidance was effective after 4.5 years.
2. Uptake for a liver rescan after 4.5 years.
3. Whether the time interval of 4.5 years between liver scans is effective in monitoring the progression of liver fibrosis and to report the change in liver fibrosis stage between baseline and follow-up scans.

## Method

### Design

**Figure 1.**
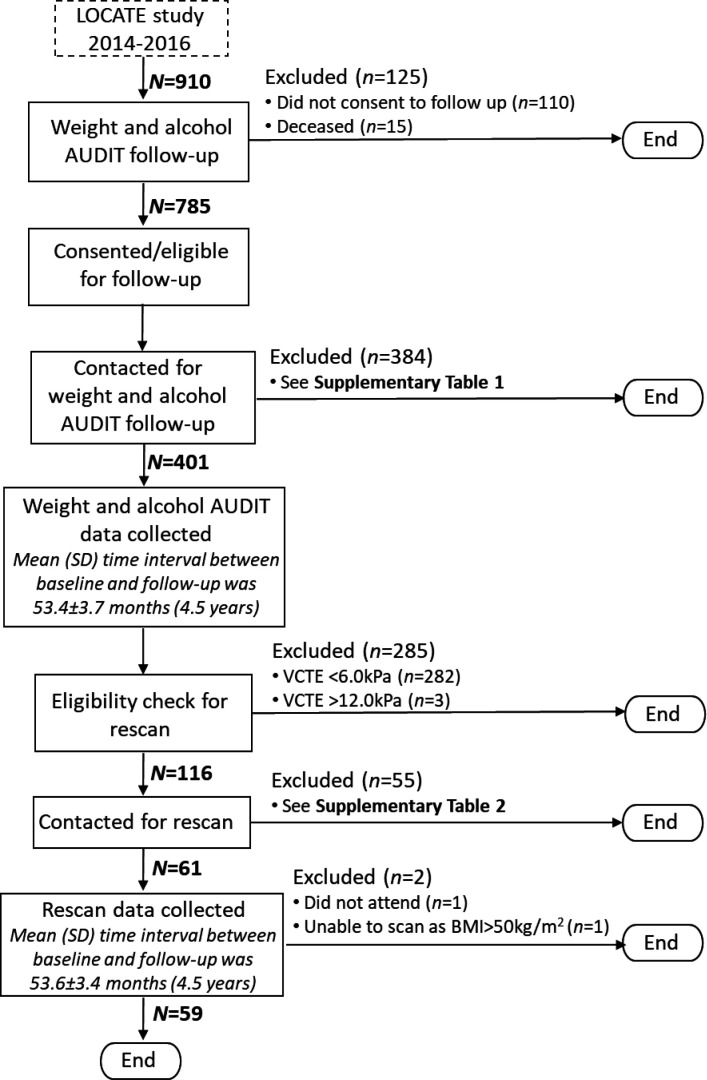
Flow of participants through the LOCATE follow-up study. AUDIT = Alcohol Use Disorders Identification Test. BMI = body mass index. VCTE = vibration-controlled transient elastography.

This was a follow-up study after the LOCATE intervention. The study design and methods of the LOCATE intervention have been reported previously.^13^ Figure 1 shows the flow of participants.

### Measurable outcomes

The measurable outcomes were as follows:

1. Change in alcohol AUDIT grade between baseline and follow-up using the World Health Organization AUDIT questionnaire.^15,16^
2. Change in weight (kg) between baseline and follow-up.
3. Uptake of patients attending for liver rescanning.
4. Change in liver fibrosis between baseline and follow-up, measured using VCTE.
5. The proportion of patients whose liver fibrosis stage has progressed between baseline and follow-up.

### Procedure

The LOCATE database of patients (*n* = 910) was screened to exclude deceased patients (*n* = 15) and patients who had declined to be contacted for follow-up (*n* = 110). The remaining patients (*n* = 785) were telephoned between August 2019 and May 2020, and invited to take part in the follow-up. Patients who agreed to take part (*n* = 401) were asked to report their current weight and answer alcohol AUDIT questions.

After the weight and alcohol AUDIT follow-up, all eligible patients were invited for a repeat liver scan using the parameters below.

#### Exclusion criteria

Patients whose baseline VCTE readings were <6.0 kPa (*n* = 282) and ≥12.0 kPa (*n* = 3) were excluded. See Supplementary Box 4 for further details on the exclusion criteria.

#### Inclusion criteria

Patients with a baseline VCTE reading of ≥6.0 kPa and <12.0 kPa (*n* = 116) were included. See Supplementary Box 5 for further details on the inclusion criteria.

Two recruitment methods were used to invite patients for a rescan:

1. The study team wrote to the GPs of all patients eligible for a rescan to ask them to refer their patients to the community liver service (Supplementary Box 6).
2. The study team also telephoned all eligible patients directly to invite them for a rescan.

Rescans took place at two primary care sites in Southampton. The FibroScan Mini +430 and 402 models were used. All patients who had a rescan were informed of their follow-up reading and how it compared with their baseline scan. All patients, except one, consented for their GP to be advised of the repeat liver scan reading (Supplementary Box 7). Patients whose follow-up VCTE reading was >10.0 kPa were referred to a secondary care hepatology clinic, as per the locally agreed referral pathway.^17^

### Analysis

The alcohol AUDIT scores, weight of patients, and VCTE readings were analysed using SPSS statistics software (version 27).

At the time of the LOCATE final study, validated VCTE cut-off values for each of the stages of liver fibrosis were not well established. At follow-up, validated cut-off values were used from the results of a large biopsy study published in 2019,^18^ see Supplementary Table 3 for a comparison of cut-off values.

A 15% coefficient of variation was applied to the rescan readings to reliably identify any changes to fibrosis stage between baseline and follow-up.^19^ Standard descriptive statistics were used to summarise variables: mean (SD) for continuous variables or median (interquartile range [IQR]) for skewed variables, and numbers and percentages for categorical variables. Paired samples *t*-tests were used to determine the mean differences between baseline and follow-up. The χ^2^ test of independence (*α* = 0.05) was used to determine the relationship between categorical variables. A two-tailed independent samples *t*-test was used to compare the differences between groups, and a binary logistic regression analysis was used to test the relationship between the baseline independent variables and the outcome of liver fibrosis stage progression at follow-up.

## Results

Mean (SD) time interval between baseline and the weight and alcohol AUDIT follow-up was 53.4±3.7 months.

Baseline characteristics from patients who took part in the weight and alcohol AUDIT *(* *n* = 401) were analysed and compared with all patients who consented to be contacted for follow-up (*n* = 785). It was found there were no differences in sex, ethnic group, body mass index (BMI), and weight. The median (IQR) age of patients who completed the weight and alcohol AUDIT follow-up was higher than the overall cohort of patients who consented to take part in the follow-up: 52 (40–60) years and 51 (39–60) years, respectively (*P* = 0.009). Patients with ‘high’ alcohol AUDIT grades were less likely to take part in the weight and alcohol AUDIT follow-up than patients with a ‘low risk’ alcohol AUDIT grade (*P* = 0.016). (Supplementary Table 4).

Baseline and follow-up characteristics of patients who took part in the weight and alcohol AUDIT questions (*n* = 401) were analysed and compared. At follow-up the median (IQR) BMI was lower than at baseline, respectively: 28.0 (40–60) kg/m^2^ and 28.1 (24.8–33.1) kg/m^2^ (*P* = 0.008). Mean (SD) weight loss was 1.2 kg±8.4 kg (*P* = 0.005), and, when compared with the baseline, patients were more likely to have a ‘high’ alcohol AUDIT grade than a ‘low risk’ alcohol AUDIT grade (*P* = <0.001) (Supplementary Table 5).

50.9% (*n* = 59) of participants eligible for a rescan (*n* = 116) accepted the invitation and underwent a liver rescan (Supplementary Table 2). Their characteristics were analysed and compared with all patients who were eligible to take part in the rescan follow-up. The study found there to be no differences in sex, ethnic group, T2DM, age, BMI, weight, fibrosis stage, and alcohol AUDIT grades (Supplementary Table 6).

Mean (SD) time interval between baseline and follow-up scans was 53.6±3.4 months (4.5 years).

When compared with baseline, there was no change to fibrosis stage at follow-up for 32.2% of patients (*n* = 19) and a decrease in fibrosis stage in 49.1% of patients (*n* = 29). At follow-up, it was found 18.7% (*n* = 11) of patients’ liver fibrosis stage had progressed: 3.4% (*n* = 2) to F2 (8.2 kPa–9.6 kPa); and 15.3% (*n* = 9) to F3 (9.7 kPa–13.5 kPa) and F4 (≥13.6 kPa) (Table 1 and Supplementary Table 7).

**Table 1.** Summary of patient VCTE fibrosis stage changes between baseline and follow-up scans (*n* = 59)

**Change in fibrosis stage**
Significant change (F1 to F2)^a^ (*n*, %)	2	3.4	Progressors (*n* = 11, 18.7%)
Advanced change (F1, F2, F3 to F3, F4)^b^ (*n*, %)	9	15.3	
No change (*n*, %)	19	32.2	Non-progressors(*n* = 48, 81.3%)
Decrease (*n*, %)	29	49.1	

VCTE = vibration-controlled transient elastography. ^a^6.0 kPa–8.1 kPa to 8.2 kPa–9.6 kPa. ^b^6.0 kPa–8.1 kPa, 8.2 kPa–9.6 kPa, 9.7 kPa–13.5 kPa to (9.7 kPa–13.5 kPa), ≥13.6 kPa

The characteristics of patients whose liver fibrosis had progressed (’progressors’) were compared with patients whose liver fibrosis had remained the same or reversed (‘non-progressors’), and it was found there were trends towards increased BMI and increased proportions with T2DM among participants who experienced progression of their liver disease. The mean ± SD change in kPa for the ‘progressors' was 6.4±3.5 kPa, and for the 'non-progressors' was –1.5±2.0 kPa (*P* = 0.041) (Table 2).

**Table 2.** Characteristics of patients who, at follow-up, had either progressed their liver fibrosis stage (progressors) or their liver fibrosis stage had remained the same or reversed (non-progressors)

Characteristics	**Progressors**(***n* = 11, 18.7%**)	**Non-progressors**(***n* = 48, 81.3%**)	***P* value**
Male sex, *n* (%)	6	54.5	32	66.7	0.478^a^
Median age, years (IQR)	58	48–67	57	51–65	0.917^b^
BAME ethnic group*, n* (%)	2	18.2	8	16.7	0.927^a^
T2DM positive*, n* (%)	9	81.8	25	52.1	0.083^a^
Fibroscan readings:					
Mean baseline kPa (SD)	7.2	0.8	7.7	1.7	0.024^b^
Mean follow-up kPa (SD)	13.6	3.8	6.1	1.9	0.007^b^
Mean change in kPa between baseline and follow-up (SD)	6.4	3.5	–1.5	2.0	0.041^b^
BMI (kg/m^2^)					
Median baseline (IQR)	33.6	28.5–39.4	32.0	27.5–35.4	0.189^b^
Median follow-up (IQR)	33.3	28.7–37.3	30.5	26.3–36.6	0.706^b^
Mean time interval between scans, months (SD)	52.5	2.9	53.9	3.4	0.164^b^
Alcohol AUDIT grade:^c^					
Baseline high, *n* (%)^d^	2	18.2	16	33.3	0.287^a^
Follow-up high, *n* (%)^d^	4	36.4	18	38.3	0.866^a^

^a^ *P* values refer to a χ2 test of independence using an alpha level of 5%^. b^ *P* values refer to a two-tailed independent samples *t*-test using a confidence interval of 95%. ^c^Two patients are excluded from the alcohol AUDIT grade change as they declined to complete the questionnaire at follow-up. ^d^High = hazardous, harmful, and dependent alcohol AUDIT grades. BAME = Black and minority ethnic. BMI = body mass index. T2DM = type 2 diabetes mellitus.

Two separate binary logistic regression analyses were undertaken to investigate whether any of the measured baseline factors were independently associated with (a) the progression of liver fibrosis stage to F3 (9.7 kPa–13.5 kPa) or F4 (≥13.6 kPa); and (b) the regression or no change of liver fibrosis stage to F2 (8.2 kPa–9.6 kPa), F1 (6.0kPa – 8.1kPa), or F0 (<6.0 kPa). These data showed that none of the factors were associated with the progression or regression or ‘no change’ of liver fibrosis, although it should be noted that the study lacks sufficient power to adequately test this important question. The model included T2DM, age, sex, baseline VCTE reading, baseline BMI, and alcohol AUDIT grade and is shown in Supplementary Tables 8 and 9.

## Discussion

### Summary

**Figure 2.**
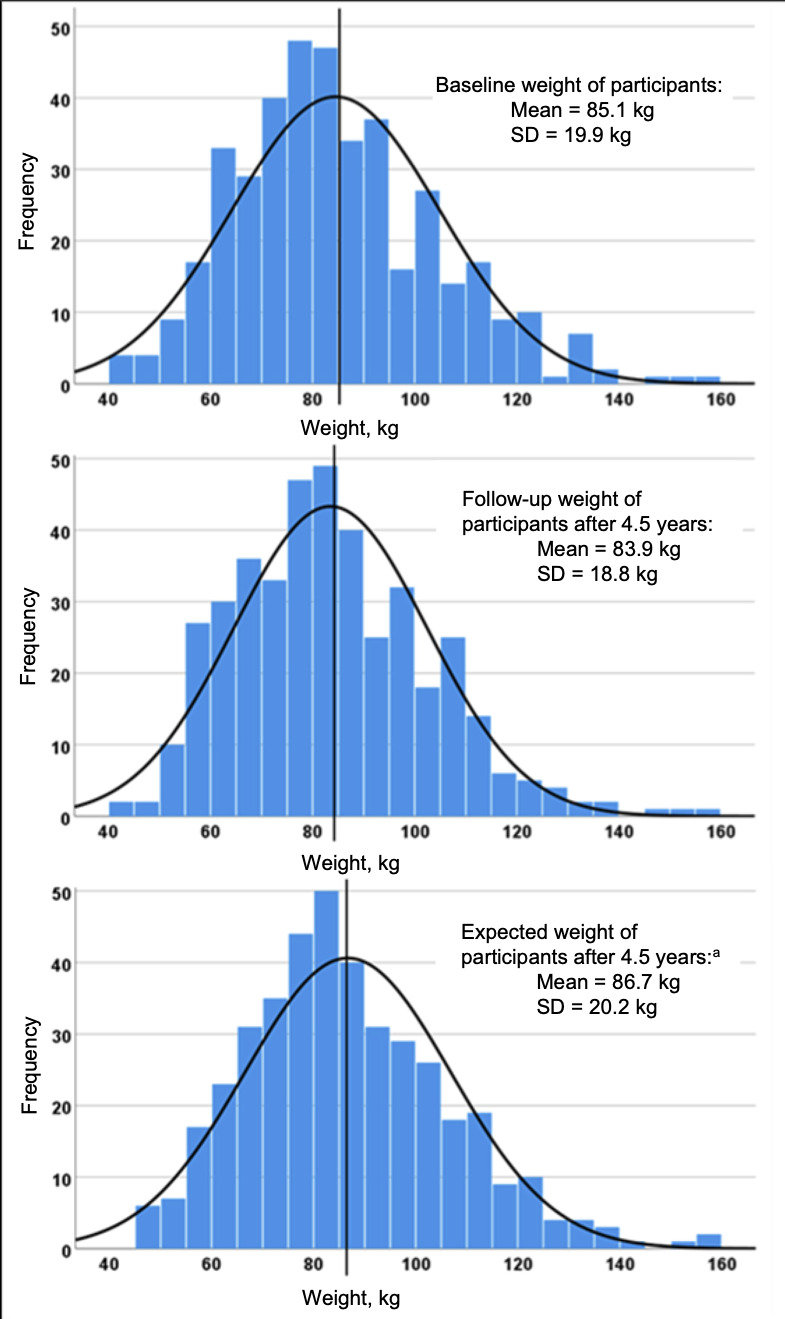
Comparison of the distribution of participants’ weight at baseline, follow-up and ‘expected’ follow-up weight (calculated from the weight gain per annum observed in recent [2020] Public Health England data).^20^ ^a^The period between baseline and follow-up was 4.5 years. To calculate the expected weight of participants at follow-up, the authors added 0.5kg^1^ x 4.5 years to the baseline weight of participants who were ≥40 years old at baseline. For participants who turned 40 during the follow-up period, 0.5kg per year was added pro rata.

The results show that standard care advice regarding weight loss and alcohol consumption has, after 53.4±3.4 months (mean ± SD), had little effect on weight and alcohol consumption. In the UK, likelihood of overweight and obesity tends to increase with age.^20^ The findings suggest that the participants followed-up in the study have arrested this trend. They lost an average of 1.2 kg in body weight, while the UK data suggest that, without the BI, they would have gained an average of 1.6 kg during the period of follow-up.^20^ Thus, with the important caveat that the study did not have a control group, it is possible to interpret the data, as participants having achieved a mean net weight loss of 2.8 kg during the study (see Figure 2). That said, although weight gain has been arrested, weight loss of 1.2 kg or 1.4% is very small, and such a small percentage weight loss is of questionable clinical significance for improving liver disease.

There was a limited response to the invitation for a rescan (50.9%), and evidence of advanced progression of liver disease after 53.6±3.4 months (mean ±SD) was identified in 15.3% of patients whose fibrosis stage had progressed from F1 (6.0 kPa–8.1 kPa) or F2 (8.2 kPa–9.6 kPa) to F3 (9.7 kPa–13.5 kPa) or F4 (≥13.6 kPa). None of the baseline factors were independently associated with progression to F3 (9.7 kPa–13.5 kPa) or F4 (≥13.6 kPa) fibrosis.

### Strengths and limitations

To the authors' knowledge, this is the first study based in primary care that has used VCTE to follow-up on patients who were determined on baseline scanning to be ‘at risk’ of progression of liver fibrosis. It has been shown that a single simple intervention with standard care advice regarding weight management and alcohol consumption did not have a substantial effect on weight management or alcohol consumption after 53.4 months. Additionally, estimates have been provided of likely progression of liver disease in primary care patients whose liver disease was not deemed to be sufficiently severe to warrant referral to the secondary care hepatology service. In those patients consenting to a rescan after 53.6 months, the data show that there was progression of liver disease in 15.3% of patients whose fibrosis stage had progressed from mild or significant fibrosis (F1 [6.0 kPa–8.1 kPa] and F2 [8.2 kPa–9.6 kPa]) to advanced fibrosis or cirrhosis (F3 [9.7 kPa–13.5 kPa] and F4 [≥13.6 kPa]). This indicates that there is a need to establish a rescan service more widely in primary care, and there is also a need to better identify those individuals at baseline in this setting who are at risk of liver fibrosis progression.

Limitations of this study include the loss to follow-up, which was disappointing. The follow-up data were collected during the COVID-19 pandemic, which included intermittent periods of restriction on movement in the UK. It is possible that there is regression to the mean with repeat scanning; however, the inclusion criteria for rescanning did not include participants at the extreme ends of the distribution curve (only participants with baseline scan results of ≥6.0 kPa and <12.0 kPa were included). Thus, the effect of regression to the mean in this cohort is likely to be small. Additional limitations were: the follow-up cohort was a predominantly a White ethnic group; the authors were unable to verify the T2DM status for a significant number of the patients who took part in the weight and alcohol AUDIT follow-up; and patients self-reported their weight.

### Comparison with existing literature

Patient adherence to lifestyle-based interventions is often poor and presents a significant challenge.^21^ Current evidence indicates that lifestyle modifications to lose weight will improve liver health,^22–24^ yet the present study has shown that current standard care lifestyle advice does not lead to persistent change. A recommendation would be to provide healthcare professionals with additional resources, such as behavioural strategies, which have been demonstrated to improve patient adherence to lifestyle changes; for example, self-monitoring^25^ (where participants keep a record of, for example, their food and alcohol intake); treatment tailoring^26^ (making flexible treatment recommendations for individual preferences); social support^27,28^ (including family members or offering group-based support); skills training^29^ (training participants to problem solve); and extended care^25^ (where long-term contact is maintained).

Other health screening services (for example, bowel) have found that patients of low socioeconomic status (SES) are less likely to participate.^30^ To address this, the authors recommend that GPs are given the support to promote awareness and knowledge of liver disease,^31,32^ particularly in low SES settings and in patients with obesity or T2DM,^33^ and better educational resources are made available to patients.^34^ There is limited evidence at present regarding the prognosis of those identified with liver disease in the community; the present study adds to the evidence base.

### Implications for practice

This study found that, unsurprisingly, further support is required to help patients make lifestyle changes. With additional resources, primary care can play an important role in helping patients to make positive sustainable lifestyle changes. This does not have to increase the workload of primary care physicians. For example, in Southampton there is a community liver service for GPs to refer patients to if they suspect they may have liver disease. This service is funded by the clinical commissioning group (CCG) and, at the time of the liver assessment, patients have a 20-minute discussion focused on behaviour change where potential lifestyle changes are discussed. Losing weight and reducing alcohol consumption within UK guidelines are not just recommended to improve liver health, they will also lower the risk factors associated with malignancy, T2DM, coronary heart disease, and chronic kidney disease.^6,35^

Any commissioning team considering implementing a liver screening service in primary care to identify liver disease should look at developing effective strategies to improve uptake, such as better educational resources for patients,^36^ GP endorsement, and personalised reminders for non-participants.^32^

Importantly, this study has highlighted that patients identified with intermediate fibrosis levels in community screening programmes are at moderate risk of progression, and robust follow-up and engagement is needed to maintain contact. If there are no specific factors (for example, continued high alcohol consumption) that suggest patients will rapidly progress from mild fibrosis to advanced fibrosis and cirrhosis, in the authors' opinion, the recommendation should be to manage patients on the basis that their liver disease will progress to advanced fibrosis and cirrhosis.

## Trial registration number

Trial registration number: 13/SC/0012; post-results. LOCATE - LOcal Care And Treatment Evaluation (ISRCTN 03563761) https://doi.org/10.1186/ISRCTN03563761.
